# Conductive Particles Enable Syntrophic Acetate Oxidation between *Geobacter* and *Methanosarcina* from Coastal Sediments

**DOI:** 10.1128/mBio.00226-18

**Published:** 2018-05-01

**Authors:** Amelia-Elena Rotaru, Federica Calabrese, Hryhoriy Stryhanyuk, Florin Musat, Pravin Malla Shrestha, Hannah Sophia Weber, Oona L. O. Snoeyenbos-West, Per O. J. Hall, Hans H. Richnow, Niculina Musat, Bo Thamdrup

**Affiliations:** a Department of Biology, University of Southern Denmark, Odense, Denmark; b Helmholtz Centre for Environmental Research, Leipzig, Germany; c Energy Bioscience Institute, University of California Berkeley, Berkeley, USA; d Department of Marine Sciences, University of Gothenburg, Gothenburg, Sweden; Oregon State University

**Keywords:** *Desulfuromonadales*, *Geobacter*, *Methanosarcina*, nanoSIMS, activated carbon, competitive exclusion, direct interspecies electron transfer, syntrophic acetate oxidation

## Abstract

Coastal sediments are rich in conductive particles, possibly affecting microbial processes for which acetate is a central intermediate. In the methanogenic zone, acetate is consumed by methanogens and/or syntrophic acetate-oxidizing (SAO) consortia. SAO consortia live under extreme thermodynamic pressure, and their survival depends on successful partnership. Here, we demonstrate that conductive particles enable the partnership between SAO bacteria (i.e., *Geobacter* spp.) and methanogens (*Methanosarcina* spp.) from the coastal sediments of the Bothnian Bay of the Baltic Sea. Baltic methanogenic sediments were rich in conductive minerals, had an apparent isotopic fractionation characteristic of CO_2_-reductive methanogenesis, and were inhabited by *Geobacter* and *Methanosarcina*. As long as conductive particles were delivered, *Geobacter* and *Methanosarcina* persisted, whereas exclusion of conductive particles led to the extinction of *Geobacter*. Baltic *Geobacter* did not establish a direct electric contact with *Methanosarcina*, necessitating conductive particles as electrical conduits. Within SAO consortia, *Geobacter* was an efficient [^13^C]acetate utilizer, accounting for 82% of the assimilation and 27% of the breakdown of acetate. *Geobacter* benefits from the association with the methanogen, because in the absence of an electron acceptor it can use *Methanosarcina* as a terminal electron sink. Consequently, inhibition of methanogenesis constrained the SAO activity of *Geobacter* as well. A potential benefit for *Methanosarcina* partnering with *Geobacter* is that together they competitively exclude acetoclastic methanogens like *Methanothrix* from an environment rich in conductive particles. Conductive particle-mediated SAO could explain the abundance of acetate oxidizers like *Geobacter* in the methanogenic zone of sediments where no electron acceptors other than CO_2_ are available.

## IMPORTANCE

Acetate-oxidizing bacteria are known to thrive in mutualistic consortia in which H_2_ or formate is shuttled to a methane-producing *Archaea* partner. Here, we discovered that such bacteria could instead transfer electrons via conductive minerals. Mineral SAO (syntrophic acetate oxidation) could be a vital pathway for CO_2_-reductive methanogenesis in the environment, especially in sediments rich in conductive minerals. Mineral-facilitated SAO is therefore of potential importance for both iron and methane cycles in sediments and soils. Additionally, our observations imply that agricultural runoff or amendments with conductive chars could trigger a significant increase in methane emissions.

## INTRODUCTION

Syntrophic acetate-oxidizing (SAO) bacteria live in a mutualistic interaction with methanogenic archaea, which feed on the H_2_ or formate released by the SAO bacterial partner (1). Besides H_2_ or formate, cysteine can also be used to transfer electrons in some SAO consortia (2). Several studies with synthetic consortia have shown SAO activity in members of the phyla *Firmicutes* (*Thermacetogenium*, *Clostridium*, *Thermotoga*, *Candidatus* Contubernalis, and *Syntrophaceticus*) and *Proteobacteria* (*Desulfomicrobium* and *Geobacter*) (2–14). Remarkably, acetoclastic methanogens (*Methanosarcina* and *Methanothrix*) have been proposed to play the role of syntrophic acetate oxidizers when provided with an appropriate H_2_-consuming partner (15, 16). Some of the genera above have been suggested to carry out SAO in thermophilic digesters (17–26), lake/river sediments (21, 27, 28), tropical wetland soil (29), rice paddies (30–32), or oil field reservoirs (33). Many of these environments are rich in (semi)conductive minerals like magnetite (34, 35), pyrite (36, 37), or black carbon resulting from incomplete burning of plant biomass (38–40). Electrically conductive iron oxide minerals and carbon chars (magnetite, granular activated carbon, biochar) were previously shown to stimulate direct interspecies electron transfer (DIET), a recently described form of interspecies electron transfer (12, 41–49), whereas strict H_2_-based interactions were shown to remain unaffected by the addition of conductive materials (44). DIET is a syntrophic association where electrons are transferred via conductive and/or redox-active cell surface structures between an electron-donating species (electrogen) and an electron-accepting species (electrotroph) (47–49). Conductive minerals seem to alleviate the need for cells to produce certain cell surface molecules required for DIET (41). DIET mediated by conductive materials is considered a novel strategy to stimulate recalcitrant organic matter decomposition in anaerobic digesters (50–52) and to enhance methanogenic decomposition of organics in rice paddies (46, 53) and aquatic sediments (28, 54). It is likely that conductive materials replace the molecular conduits that cells require to establish direct contacts during DIET.

Although SAO via DIET is considered thermodynamically favorable at pH values between 1.9 and 2.9 and impossible at pH 7 (55), conductive minerals have been shown to facilitate SAO in synthetic denitrifying consortia at pH 7 (56). Nevertheless, the impact of minerals on environmentally relevant SAO is presently not understood. Mineral-facilitated SAO (here called mineral-SAO) could be significant in coastal environments rich in (semi)conductive minerals (36, 57–59). Such (semi)conductive minerals are likely to impact microbial processes (36, 56), for which acetate is a central intermediate (88–90).

Here, we investigated the role of mineral-SAO in methanogenic processes from coastal sediments. We examined if electrically conductive materials mediate SAO between *Geobacter* and *Methanosarcina* organisms coexisting in the brackish, iron-rich coastal sediments of Bothnian Bay. Our results indicate that mineral-SAO may impact both the iron and the methane cycles in these sediments, with implications for atmospheric methane emissions.

## RESULTS AND DISCUSSION

In this study, we found that methanogenic communities from Bothnian Bay made use of (semi)conductive particles to facilitate SAO. For this, we used a combination of physiological and stable isotope labeling experiments followed by monitoring of labeled products and incorporation of the labeled substrate in phylogenetically assigned cells by using nanoscale secondary ion mass spectrometry (nanoSIMS) coupled with catalyzed reporter deposition fluorescent *in situ* hybridization (CARD-FISH).

Syntrophic acetate oxidizers are difficult to enrich (57), because SAO is thermodynamically challenging (55). Here, we successfully enriched SAO consortia from temperate sediments (sediment temperature, 15°C; incubation temperature, 20 to 25°C) by successive cultivation in the presence of electrically conductive (>1,000 S/m [58]) granular activated carbon (GAC).

### Characteristics of the Bothnian Bay methanogenic zone. (i) Geochemistry.

Our hypothesis was that a high conductive mineral content would stimulate electric interactions between abundant electroactive microorganisms coexisting in the methanogenic zone. The Bothnian Bay sediments are rich in conductive minerals dispersed either within the fine structure of sediments or within ferromanganese nodules (59).

**FIG 1:**
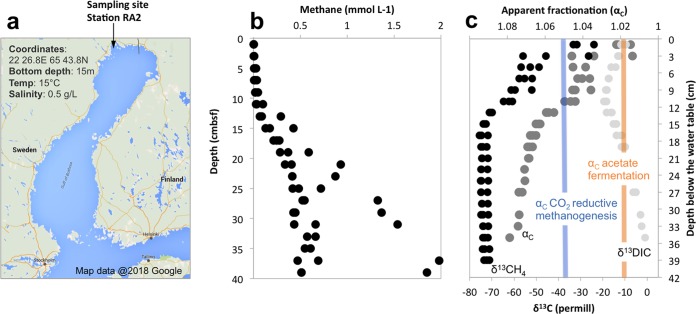
CO_2_-reductive methanogenesis in the Bothnian Bay methanogenic zone. (a) The sampling site, RA2, was located off the Bothnian Bay northern coast. (b and c) Here, methane accumulated close to and sometimes over the saturation limit (b) and was strongly depleted in ^13^C (low δ^13^CH_4_), which indicated a high apparent fractionation (α_C_) characteristic of CO_2_-reductive methanogenesis (c). Previous studies showed an α_C_ of ca. 1.05 (blue line) in *Methanosarcina* grown via CO_2_-reductive methanogenesis (85, 86). An α_C_ of ca. 1.02 (orange line) was observed in *Methanosarcina* species grown by acetoclastic methanogenesis (87).

To explore mineral-mediated interactions in Bothnian Bay, we sampled the methanogenic zone of these sediments to verify the mineral content. Sediment cores were collected from 15-m water depth at station RA2, located at 65°43.6′N and 22°26.8′E in Bothnian Bay (Fig. 1), which had high sediment temperature (15°C) and low *in situ* salinity (0.5). The mineral content was low in manganese oxides (13 ± 3 µmol/cm^3^ [mean ± standard deviation] from both HCl and dithionite extractions), high in FeS, FeCO_3_, and other poorly crystalline Fe-minerals (229 ± 8 µmol/cm^3^), and high in crystalline iron oxides (dithionite-extractable iron, 131 ± 4 µmol/cm^3^) and conductive magnetite (32 ± 7 µmol/cm^3^ oxalate extractable). This estimate of the magnetite content was similar to what has been previously observed below the sulfate-methane transition zone in Baltic Sea sediments (ca. 30 µmol/cm^3^) (60).

Besides iron oxide minerals, previous studies showed that black carbon, also a conductive material (40), dominated the coastal sediments of the Baltic Sea, representing 1.7% to 46% of the total organic carbon (TOC) in sediments closer to coastal towns (61). Conductive materials could reach Bothnian Bay by river runoff from the eight rivers entering the bay from Sweden and Finland, and also via runoff from the forestry industry and various coastal industries (59, 62).

The high abundance of conductive particles likely stimulates electrical interactions between abundant electroactive microorganisms that coexist in the methanogenic zone (41–43, 45, 52). Methane reached its highest concentrations below 25 cm depth (Fig. 1). In the methanogenic zone, two independent processes, SAO and/or acetoclastic methanogenesis, could consume acetate, a key intermediate of organic matter decomposition. SAO bacteria would need a CO_2_-reductive methanogenic partner to scavenge the electrons released during acetate oxidation. To find out if CO_2_-reductive methanogenesis was occurring in these sediments, we looked at the apparent isotopic fractionation of dissolved organic carbon (DIC, which includes CO_2_, carbonic acid, bicarbonate, and carbonate) and methane. Methane was strongly depleted in ^13^C relative to DIC (median δ ^13^CH_4_, −74‰, median δ ^13^DIC, −2.5‰) (Fig. 1), which resulted in a signature apparent isotopic fractionation (α_c_) of 1.07, characteristic of CO_2_-reductive methanogenesis (63).

### (ii) Microbial community.

**FIG 2:**
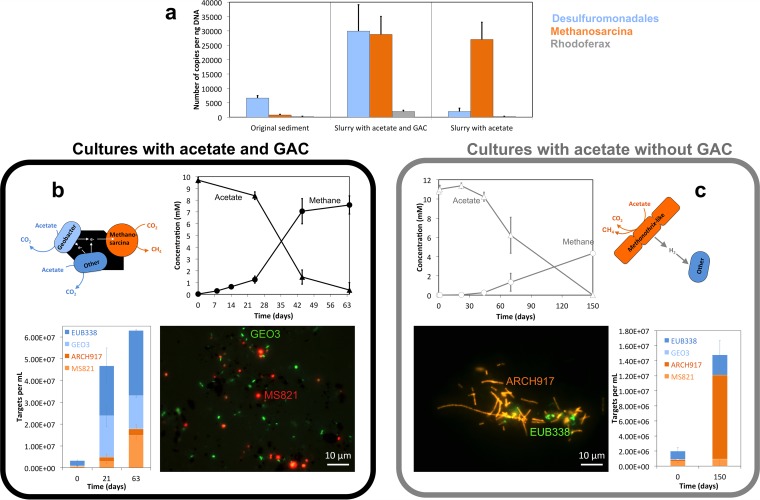
Incubation mixtures with and without activated carbon and representative organisms. (a) Quantitative PCR in original sediment samples showed that *Desulfuromonadales* were the dominant electrogens in the original sediment and in sediment slurries with conductive particles, but this group was almost extinct in a first slurry transfer without conductive particles. The only methanogens detected by qPCR in the original sediments were DIET-associated *Methanosarcina*, which remained abundant in slurry incubation mixtures with or without conductive particles. (b) In mud-free incubation mixtures with conductive GAC (sixth consecutive mud-free transfer), acetate was completely depleted after 63 days, and it was converted to methane with a high stoichiometric recovery (82%). *Methanosarcina* was the only *Archaea* genus detected in these mud-free cultures. Together, *Methanosarcina* and *Geobacter* represented ca. half of the microbial community, as determined by CARD-FISH. (c) On the other hand, in control incubation mixtures without conductive materials (third consecutive mud-free transfer), acetate consumption was much slower. Acetate was depleted after 150 days and converted to methane, with only 40% stoichiometric recovery. In control incubation mixtures without conductive GAC, *Geobacter* and *Methanosarcina* were led to extinction (Fig. S5F). Instead *Methanothrix*-like filamentous *Archaea* carried acetate utilization in control incubation mixtures without GAC (Fig. S5F).

DIET consortia (*Geobacter* and *Methanosarcina*) can usually form more efficient electron transfer associations via conductive minerals than they do in their absence (42–44, 64). In contrast, H_2_-transferring consortia have been shown to remain little affected by conductive materials (44, 65). We predicted that Bothnian Bay sediments rich in conductive minerals are favorable for mineral DIET associations. As anticipated, these iron mineral-rich sediments harbored *Proteobacteria*, including exo-electrogens related to *Geobacter* and *Rhodoferax*, and *Archaea* methanogens related to *Methanosarcina* (Fig. 2a; see also Fig. S2F). Both *Geobacter* and *Rhodoferax* were previously shown to form DIET associations with species of Methanosarcinales (48, 64; A.-E. Rotaru and D. R. Lovley, unpublished data). Until now, only Methanosarcinales were shown to establish DIET associations with electrogens (48, 49, 64), probably due to their high *c*-type cytochrome content, which allows for electron uptake from electrogens (48, 66).

10.1128/mBio.00226-18.2 FIG S1  Numbers of reads were sufficient in all three sediment cores, as indicated by the flattening rarefraction curves (a) and a similar pattern for the rare operational taxonomic units (OTUs) estimator, Chao1 (b). Download FIG S1, PDF file, 0.2 MB. Copyright © 2018 Rotaru et al. 2018 Rotaru et al. This content is distributed under the terms of the Creative Commons Attribution 4.0 International license.

10.1128/mBio.00226-18.3 FIG S2  16S amplicon sequencing (a) showed that *Deltaproteobacteria* (including *Geobacter*) were the most abundant *Proteobacteria*, and (b) DIET-capable Methanosarcinales and the H2-dependent methanotroph *Methanomassilicoccus* were the dominant methanogens. The most abundant *Deltaproteobacteria* OTU had as its closest cultured relative *Geobacter* spp.,independent of the BLAST database used (NCBI or JGI). Download FIG S2, PDF file, 0.04 MB. Copyright © 2018 Rotaru et al. 2018 Rotaru et al. This content is distributed under the terms of the Creative Commons Attribution 4.0 International license.

Based on the observations that (i) sediments were high in conductive mineral content, (ii) CO_2_-reductive methanogenesis prevailed, and (iii) *Methanosarcina* and electrogens cohabited, we anticipated that mineral DIET could occur in the methanogenic zone of Bothnian Bay. We tested this hypothesis in sediment incubations with or without the addition of exogenous conductive particles.

**FIG 3:**
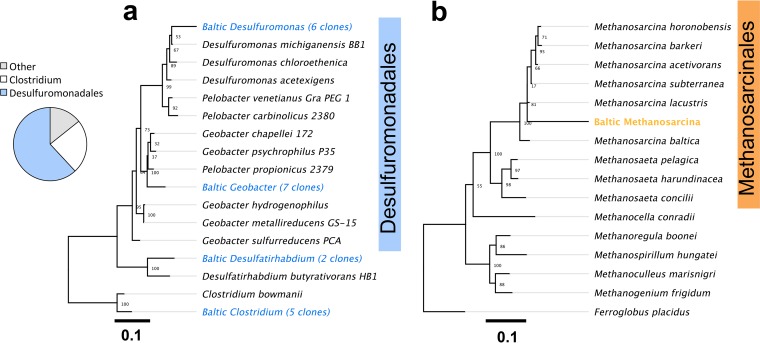
Maximum likelihood trees of *Bacteria* and *Archaea* enriched in a seventh mud-free transfer with acetate and GAC. (a) A maximum likelihood tree of representative bacterial sequences from a mud-free transfer with conductive particles (GAC), under conditions strictly promoting methanogenic respiration. Acetate-oxidizing *Desulfuromonadales* dominated the 16S rRNA clone library, with more than half displaying close relationships to Geobacter psychrophilus (97% identity) and the rest to Desulfuromonas michiganensis (98%). The only methanogens enriched on acetate and GAC were relatives of Methanosarcina subterranea (99% identity), as shown in the maximum likelihood tree in panel b.

Conductive GAC facilitated methane production from acetate (Fig. 2) and other substrates (ethanol, butyrate, and glucose) that were degraded via acetate (Fig. S3F). Tests with conductive magnetite showed that it stimulated methanogenesis even more than GAC (Fig. S4F). On the other hand, nonconductive glass beads did not facilitate methanogenesis from ethanol (Fig. 3SF), as these mixtures produced as much methane as incubation mixtures without GAC (*P* = 0.45). However, GAC was the preferred conductive particle, because we could concentrate rigorously on electron transfer (42), whereas with use of (semi)conductive magnetite (Fe^II^Fe^III^_2_O_4_) its Fe^III^ content could additionally drive iron reduction, especially during long-term incubations (67, 68).

10.1128/mBio.00226-18.4 FIG S3  Accumulation of methane and acetate in slurry incubation mixtures after 27 days of growth on different substrates. Methane (a) and acetate (b) accumulated in slurries provided with GAC (black), in controls amended with nonconductive glass beads (gray pattern), and in controls free of GAC (white). Download FIG S3, PDF file, 0.05 MB. Copyright © 2018 Rotaru et al. 2018 Rotaru et al. This content is distributed under the terms of the Creative Commons Attribution 4.0 International license.

10.1128/mBio.00226-18.5 FIG S4  Magnetite stimulates methanogenesis more than GAC. An RA2 enrichment pregrown with GAC plus acetate was transferred with conductive magnetite (red), GAC (black),or without minerals (gray, dashed). The enrichment was grown in triplicate with a 20% inoculate. Download FIG S4, PDF file, 0.03 MB. Copyright © 2018 Rotaru et al. 2018 Rotaru et al. This content is distributed under the terms of the Creative Commons Attribution 4.0 International license.

### Syntrophic acetate oxidation mediated by GAC.

Repeated transfers of the SAO cultures with acetate as electron donor, CO_2_ as electron acceptor, and GAC produced methane much faster than GAC-free controls and led to sediment-free cultures enriched in *Desulfuromonadales* (*Geobacter* and *Desulfuromonas*) and *Methanosarcina* (Fig. 2). The enriched *Desulfuromonadales* were related to acetate oxidizers like G. psychrophilus with (97% sequence identity) and D. michiganensis (98% sequence identity) (Fig. 3). The only methanogens detected in mud-free enrichments were related to Methanosarcina subterranea (99% sequence identity) (Fig. 3). In the absence of conductive minerals, *Geobacter* and *Methanosarcina* became undetectable after several mud-free transfers (Fig. 2), and a filamentous *Archaea* (a *Methanothrix-*like morphotype) took over acetate-only incubation mixtures (Fig. 2; Fig. S5F).

10.1128/mBio.00226-18.6 FIG S5  *Geobacter* targets could not be detected during nanoSIMS analyses of GAC-free cultures. *Methanosarcina* targets were also seldom detected (a) but could be detected during nanoSIMS (encircled in both the ^13^C fraction [b]) and by ^32^S, which showed the total biomass fraction (c). However, in these GAC-free incubation mixtures, *Methanothrix*-like filaments dominated (b and c), and incorporated [^13^C]acetate. *Eubacteria* (d) and *Geobacter* (e) targets could not be detected after SAO consortia were taken off conductive GAC for a single transfer. Only *Methanosarcina* cells persisted (d and e) in medium without conductive particles. Images are representative epifluorescence micrographs of 6 to 10 randomly selected fields. Download FIG S5, PDF file, 1.2 MB. Copyright © 2018 Rotaru et al. 2018 Rotaru et al. This content is distributed under the terms of the Creative Commons Attribution 4.0 International license.

**FIG 4:**
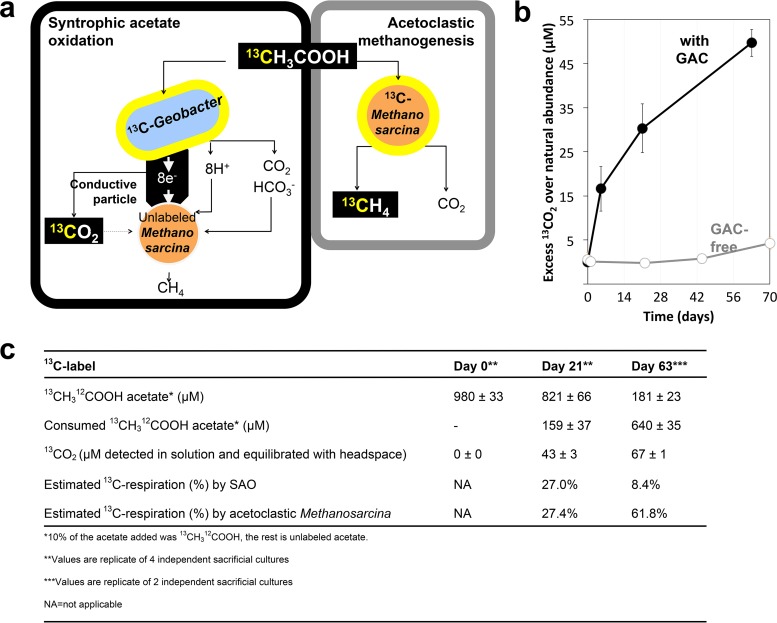
Experimental approach and evidence for SAO. (a) Experimental approach to distinguish between SAO and acetoclastic methanogenesis based on isotopic labeling. ^13^CH_3_^12^COOH was provided as 10% of the total acetate, which played the role of the electron donor for SAO consortia from the Bothnian Bay. During SAO, acetate-oxidizing *Geobacter* cells are expected to produce ^13^CO_2_ (^13^C, depicted in orange) and to incorporate [^13^C]acetate. During SAO, ^13^CO_2_ will be diluted by the bicarbonate in the medium and should not generate significant ^13^CH_4_. However, acetoclastic methanogenesis by *Methanosarcina* cells will generate ^13^CH_4_ from ^13^CH_3_^12^COOH, while cells incorporate [^13^C]acetate in their cell mass. Cells expected to incorporate [^13^C]acetate are encircled in orange. (b) SAO activity was validated by using labeled ^13^CO_2_ production from acetate, especially in SAO consortia provided with GAC (blue) versus cultures without GAC (orange). (c) An overview of acetate catabolism and how much is used for respiration by *Geobacter* versus acetoclastic methanogenesis by *Methanosarcina*.

In incubation mixtures with acetate and GAC, acetate could be consumed by acetoclastic methanogens and/or SAO consortia. A schematic representation of SAO mediated by GAC tied to methanogenesis is presented in Fig. 4. Our hypothesis was that during SAO, *Geobacter* cells donate electrons from the oxidation of acetate to GAC, which then plays the role of a transient electron acceptor. Then, *Methanosarcina* cells retrieve the electrons from GAC in order to reduce CO_2_ to methane.

To distinguish between acetoclastic methanogenesis and SAO, cultures were incubated with ^13^CH_3_^12^COOH. If acetoclastic methanogens utilized the [^13^C]methyl on acetate, they would only produce ^13^CH_4_. However, if SAO bacteria utilized [^13^C]acetate, then they would produce ^13^CO_2_ (Fig. 4). When acetoclastic methanogens and SAO bacteria use [^13^C]methyl on acetate at the same time, both ^136^CO_2_ and ^13^CH_4_ would be produced. Our results support the latter model.

### (i) SAO dependency on GAC.

Incubations for ca. 70 days with [^13^C]acetate and GAC converted the [^13^C]methyl on acetate to ^13^CO_2_, whereas control cultures lacking GAC produced little ^13^CO_2_ (Fig. 4). This indicated that indeed GAC stimulated SAO.

### (ii) Respiratory metabolism and SAO.

During exponential growth (day 21), SAO could explain 27% of the total respiratory metabolism, whereas 27.4% could be explained by acetoclastic methanogenesis (Fig. 4). During stationary phase (day 63), SAO justified 8.4% of the total respiratory metabolism, whereas acetoclastic methanogenesis justified 61.8%.

### (iii) Biosynthetic metabolism and SAO.

**FIG 5:**
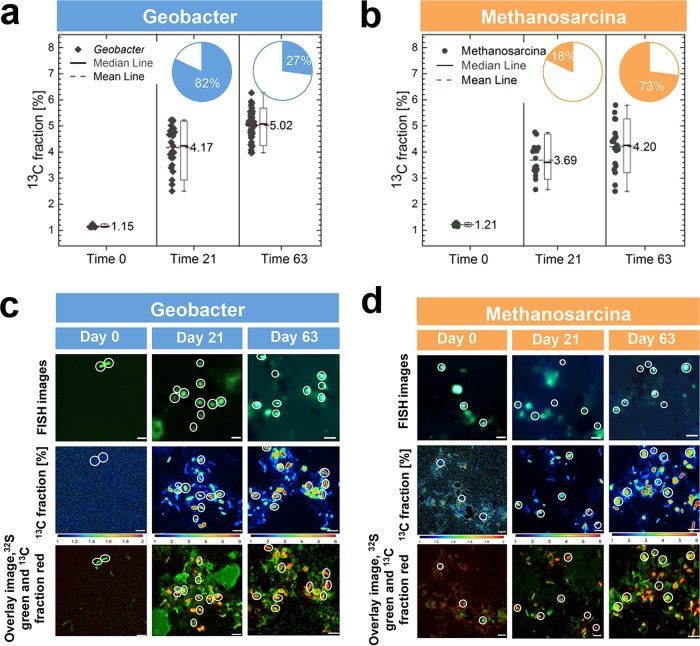
nanoSIMS identification of cells incorporating ^13^C-labeled acetate. (a and b) Highly abundant *Geobacter* cells (a) incorporated more ^13^CH_3_^12^COOH per cell than *Methanosarcina* (b). Insets for panels a and b show percent assimilation in *Geobacter* (blue insets) and *Methanosarcina* (orange) over time. (c) Time-dependent distribution of cells labeled by *Geobacter*-specific probes compared with time-dependent incorporation of ^13^CH_3_COOH in *Geobacter* cells (see scales below images) and an overlay of ^13^C incorporation (red) to total biomass as detected by tracing ^32^S (green), using nanoSIMS. (d) Time-dependent distribution of cells labeled by *Methanosarcina*-specific probes compared with time-dependent incorporation of ^13^CH_3_COOH in *Methanosarcina*-cells (see scales below images) and an overlay of ^13^C incorporation (red) to total biomass as detected by tracing ^32^S (green) using nanoSIMS.

The increase in abundance of *Geobacter* cells over time (Fig. 2) in incubation mixtures with GAC indicated that they could play the role of syntrophic acetate oxidizers in mineral-mediated SAO syntrophy. This was confirmed by analysis of the ^13^CH_3_^12^COOH-incubated SAO consortia by using nanoSIMS/CARD-FISH, an approach that helps correlate phylogeny and function (78). During incubation with GAC, both *Geobacter* and *Methanosarcina* cells became greatly enriched in ^13^C, indicating label assimilation from acetate (Fig. 5a and b). During exponential phase (day 21 *Geobacter* cells were 6 times more abundant than *Methanosarcina*) (Fig. 2). Therefore, the entire *Geobacter* population assimilated 5 times more acetate than the *Methanosarcina* population (Fig. 5). However, upon prolonged incubation (day 63), the number of *Geobacter* cells remained relatively constant, while *Methanosarcina* cells increased in abundance to match the *Geobacter* population (Fig. 2). As a consequence, during the late incubation phase, the *Methanosarcina* population assimilated 3-fold more acetate than *Geobacter* (Fig. 5).

The ratio of *Geobacter* to *Methanosarcina* cells in the original sediment (8:1) was more similar to that observed in incubation during exponential growth (6:1) than to that observed during stationary phase (1:1). During exponential growth, *Geobacter* cells incorporate a high amount of ^13^C label. Although nanoSIMS results indicated that *Geobacter* could be the primary acetate oxidizer in SAO consortia from the Baltic Sea (Fig. 5), *Desulfuromonas* might also play a significant role in the process.

### (iv) SAO is coupled to methanogenesis via a conductive particle electron conduit.

**FIG 6:**
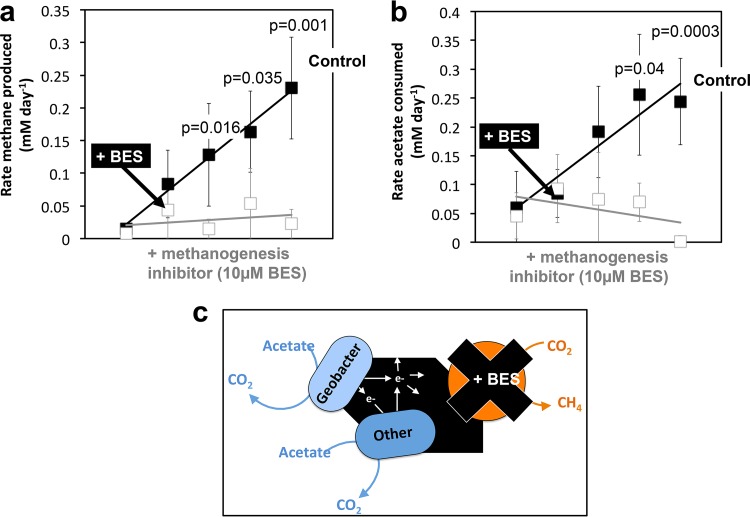
Syntrophic acetate-oxidizing bacteria cannot grow alone on acetate and GAC; they require the methanogen. If conductive GAC were sufficient for SAO bacteria to carry out acetate oxidation, the methanogenic inhibitor bromoethane sulfonate (BES) would collapse the rates of both methanogenesis (a) and acetate oxidation (b), indicating that the two processes are coupled and that *Geobacter* cannot grow alone on acetate and GAC. Methane production (a) and acetate utilization (b) rates were measured in cultures spiked with BES, in contrast to controls lacking BES and (c) a simplified representation of the BES inhibition effect on methanogenesis.

**FIG 7:**
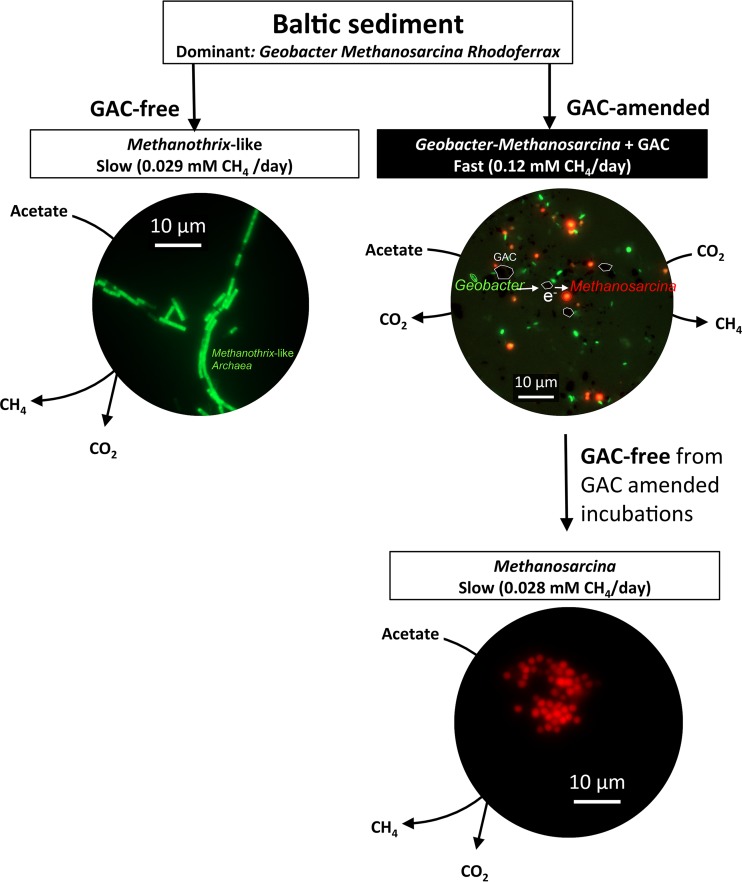
Model interactions with different treatments of a Baltic methanogenic community. *Geobacter* (green) and *Methanosarcina* (red) consortia competitively displaced *Methanothrix*-like (green) cells in Baltic sediments rich in iron-oxide minerals and in conductive particle-amended incubation mixtures. *Geobacter* was only present in incubation mixtures with conductive particles (Fig. S5F).

To verify if *Methanosarcina* was used as a terminal electron acceptor by the acetate oxidizers, we chemically inhibited the metabolic activity of the methanogen by using a methyl-coenzyme M analogue (10 µM 2-bromoethanesulfonate [BES]) (69). If the acetate oxidizers were able to respire GAC, independent of electron uptake by *Methanosarcina*, we should be able to decouple acetate utilization from methanogenesis. However, acetate utilization ceased as soon as methanogenesis was inhibited by BES (Fig. 6), indicating that the (exo)electrogenic syntrophic acetate oxidizer (*Geobacter*) used the *Methanosarcina* methanogen as an electron sink. *Geobacter*’s dependency on the methanogen could be explained either by an interspecies interaction mediated by GAC (42, 48, 64) or a direct association based on self-assembled molecular conduits on the surface of the cells (48, 49, 70). To resolve if cells adapted to carry a DIET type of interaction via redox-active surface conduits, we switched the highly enriched *Geobacter-Methanosarcina* consortia to a medium without conductive particles. Only *Methanosarcina* survived the change (Fig. 7; Fig. S5F), demonstrating that without a conductive surface, Baltic *Geobacter* could not forge connections with the methanogen on its own. This is in contrast with previous studies on synthetic *Geobacter*-*Methanosarcina* consortia (48, 64). *Geobacter*’s inability to establish an interspecies interaction with the methanogen in the absence of conductive particles suggests that *Geobacter* used the conductive particle as an electron conduit for extracellular electron transfer and *Methanosarcina* as an electron sink. In what way *Geobacter* releases electrons extracellularly onto GAC and in what way *Methanosarcina*, but not *Methanothrix*, retrieves electrons from GAC are yet unresolved. Nevertheless, the ability of *Methanosarcina* to interact with *Geobacter* via conductive particles would likely give this methanogen a competitive advantage over *Methanothrix* in mineral-rich environments like the Baltic Sea.

### (v) Exoenzymes and shuttles are not endogenously created.

Previous studies indicated that extracellular enzymes could act as manufacturers of diffusible chemicals (H_2_, formate) which could be used for electron transfer to methanogens (71). To test this hypothesis, we spiked cultures with spent medium from a fully grown culture that was filtered through a 0.2-µm filter. The spent medium should theoretically contain (exo)cellular enzymes or potential shuttles, and if these were involved in electron transfer between the microorganisms from the Bothnian Bay sediments we should see an increase in methanogenic rates. We did not notice an increase in methanogenic rates in spiked cultures compared to control cultures (Fig. S6F). This indicates that (exo)cellular enzymes/shuttles are unlikely to play a role in conductive particle-mediated SAO between *Geobacter* and *Methanosarcina*.

10.1128/mBio.00226-18.7 FIG S6  Methane production from acetate in mud-free enrichments spiked with spent filtrate (gray) or autoclaved spent filtrate (blue) versus control cultures without spent medium addition. Download FIG S6, PDF file, 0.03 MB. Copyright © 2018 Rotaru et al. 2018 Rotaru et al. This content is distributed under the terms of the Creative Commons Attribution 4.0 International license.

### Conclusion.

Here, we showed that syntrophic acetate oxidation was coupled to CO_2_-reductive methanogenesis via conductive particles in mud-free *Desulfuromonadales-Methanosarcina* consortia from the Baltic Sea. Our results suggest that conductive particles are essential for syntrophic acetate oxidation coupled to CO_2_-reductive methanogenesis in sediments. Mineral-SAO could have significant implications for the isotopic composition and the cycling of methane in aquatic sediments. Anthropogenic activity could enhance the input of conductive materials to sediments, ultimately increasing methane fluxes. Since methane is a powerful greenhouse gas, we must better understand such actuators of methane emissions in the environment.

## MATERIALS AND METHODS

### Sampling and incubations.

During an expedition on board the RV Fyrbygarrren in July 2014, we sampled sediment cores with a Gemini gravity corer. Three sediment cores were gathered at station RA2, which is located near the Swedish shoreline (coordinates: 22°26.8′E, 65°43.8′N). Within 24 h after sampling, the sediment was partitioned into depth-profiled aliquots and fixed for biogeochemical and molecular analyses inside an on-deck N_2_-inflatable glove bag, as described below in detail.

For incubations, we gathered methanogenic sediment from a depth of 30 to 36 cm and replaced the gas atmosphere with 2 × 10^5^ Pa of N_2_-CO_2_ (80:20) mix. The 30-to-36-cm-depth sediment was stored at 4°C until we generated slurries with various substrates and minerals.

Slurries were prepared in the lab in an anaerobic chamber and were generated within 6 months after sampling. For slurries, we used 3-ml cut-off syringes to distribute 2.5 ml sediment into 20-ml gas-tight vials with 7.5 ml DSM 120-modified medium. The modified DSM 120 medium was prepared as described before (48) but with 0.6 g NaCl. Sediment slurries had a high organic content, whereas mud-free enrichments did not. Therefore, we amended the mud-free enrichments with 0.2 g/liter yeast extract from a 100-g/liter anaerobic and sterile stock, which is required for methanogenic growth. Before inoculation, the complete medium which lacked the substrate and (semi)conductive minerals was dispensed anaerobically by syringe into sterile degased vials with or without minerals prepared as described below.

Conductive materials, GAC (0.1 g/10 ml; Merck), and magnetite (0.1 g/10 ml; Sigma-Aldrich) were weighed, added to vials, overlaid with 200 µl ultrapure water for wet sterilization, degased for 3 min with an N_2_-CO_2_ (80:20) mix, and autoclaved at 121°C for 25 min. Control experiments were carried out with acid-washed glass beads instead of conductive minerals. Substrates (5 mM glucose, 5 mM butyrate, 10 mM acetate, 10 mM ethanol) were added to media from sterile anoxic 1 M stocks by aseptic and anaerobic techniques. Control experiments were carried out without additional substrate to learn if the organic compounds in sediment could be used as substrates for methanogenesis. All incubations were carried out at room temperature (20 to 23°C) in triplicate unless otherwise noted.

Gas samples were withdrawn at timed intervals using hypodermic needles connected to a syringe closed by an airtight valve. Gas samples (0.5 ml) were stored, until measured, by displacing 0.5 ml ultrapure water, which filled 3-ml Exetainers. Thirty-microliter gas samples were tested for methane on a Thermo Scientific gas chromatograph equipped with a TG-Bond Msieve 5A column (30 m by 0.53 mm by 50 µm) and a flame ionization detector (FID). The carrier was N_2_ (flow rate, 5 ml/min), and we used an isothermal oven temperature of 150°C with the injector and detector set at 200°C. Gas standards (0.01% to 50% CH_4_ in N_2_) from Mikrolab Aarhus A/S were always run along with samples. Short-chain volatile fatty acids (SCVFA) were detected via high-performance liquid chromatography (HPLC) of 0.45-µm-filtered and 3-times-diluted samples. For HPLC, we used an Agilent 1100 instrument equipped with an Aminex-HPX 87H column heated at 70°C and a VWR detector, which detects SCVFA at 210 nm. Five millimoles of sulfuric acid was used as eluent at a flow rate of 0.6 ml/min. Standards used ranged between 0.1 mM and 10 mM. The detection limit for all SCVFA was 100 µM.

### Biogeochemical analyses.

To determine biogeochemical parameters, we took sediment aliquots from every 2 cm in an anaerobic glove bag filled with N_2_ gas. At this station, the sulfide-methane transition zone was below 15 cm. Geochemical parameters of direct relevance to this work were methane, dissolved inorganic carbon (DIC), and resident iron and manganese oxide species. For *in situ* methane concentrations and ^13^C/^12^C-methane isotopic fractionation, we blocked the activity of the microorganisms by immersing 2 ml active sediment into 4 ml of 2.5% NaOH. NaOH-treated samples kept in gas-tight vials were stored at 4°C, upside down, until methane could be measured.

Methane headspace concentrations were measured on a PerkinElmer gas chromatograph (GC) equipped with an EliteQPlus capillary column with an inner diameter of 0.52 mm heated to 50°C and an FID heated to 200°C. The carrier gas was N_2_ with a flow rate of 10 ml/min. δ^13^C_CH4_ values were measured at Aarhus University on an isotope-mass ratio gas chromatograph-mass spectrometer as described before (75).

For determination of iron and manganese, 5 ml of sediment was subsampled from each 2-cm-depth interval, transferred into 15-ml centrifugation vials, and stored at −20°C until extraction of the different iron and manganese phases. Three different extraction methods were applied: the cold 0.5 N HCl extraction (to dissolve poorly crystalline iron oxides FeS and FeCO_3_), the dithionite extraction (to dissolve all the other Fe-oxides except for magnetite), and oxalate extraction (to dissolve magnetite) (68, 73), followed by a ferrozine assay (74). For analysis of manganese, extractions were carried out as described for solid iron, and concentrations in the supernatant were analyzed undiluted by flame atomic absorption spectroscopy.

For pore water parameters, porosity of the sediments was calculated from identifying the relationship between the wet weight of the sediment and its dry weight. For pore water extraction, 50 ml sediment was sampled every 2 cm by scooping sediment into Falcon tubes, from which pore water was extracted with the use of rhizons (rhizosphere; pore size, 0.2 µm). Pore water work was carried out under a N_2_ atmosphere in a glove bag.

For pore water Fe^2+^ and Mn^2+^ concentrations, 1 ml pore water was mixed with 20 µl 6 N HCl and stored at −20°C. Soluble Fe^2+^ in the pore water was determined using the ferrozine assay (74).

Pore water DIC was sampled inside an N_2_-filled glove bag on board. DIC samples were filled to brim to ensure no gas bubbles into 3-ml glass vials, which contained 20 µl HgCl_2_-saturated water. Samples were stored upside down at 4°C until measurements. For measurements, we converted DIC to CO_2_ by acidification with 50 µl undiluted H_2_PO_4_ for each 200-µl DIC sample. CO_2_ was allowed to equilibrate in the headspace overnight inside 12-ml He-flushed Exetainers. DIC concentration and the [^13^C/^12^]CDIC isotope ratios were measured on an isotope ratio mass spectrometer coupled to a gas bench, as previously described (75).

### Molecular analyses.

For molecular analyses, we sampled 2 ml from every 2 cm of sediment depth. Samples were collected using cut-off syringes at the same time with samples for biogeochemical parameters, on board and inside an anaerobic bag. For safe storage during transportation, 3 depths, so a total of 6 cm, were pooled together and mixed with 6 ml MoBio RNAlater (1:1). Prior to DNA extractions, RNAlater was removed by centrifugation. For DNA extraction, we used the MoBio RNA soil kit coupled to a cDNA soil kit and followed the instructions provided by the kit manufacturer. DNA was quantified using a Nano Drop before downstream applications.

### Quantitative PCR.

To target electrogenic microorganisms, genus/order-specific PCR was performed with primers for *Desulfuromonadales* (includes all *Geobacter*), *Geothrix*, *Rhodoferrax* and *Shewanella*. For methanogens, the following genus/order-specific primers were tested to target: Methanosarcinaceae, *Methanothrix*, Methanococcales*, Methanobacteriales*, Methanomicrobiales. A list of all the primers used, making of standards, and the conditions for quantitative PCR (qPCR) are available in Table S1F and Text S1, respectively.

10.1128/mBio.00226-18.1 TEXT S1  Additional information regarding our methods. Download TEXT S1, DOCX file, 0.2 MB. Copyright © 2018 Rotaru et al. 2018 Rotaru et al. This content is distributed under the terms of the Creative Commons Attribution 4.0 International license.

10.1128/mBio.00226-18.8 TABLE S1  Primers used in this study. Download TABLE S1, DOCX file, 0.1 MB. Copyright © 2018 Rotaru et al. 2018 Rotaru et al. This content is distributed under the terms of the Creative Commons Attribution 4.0 International license.

### 16S rRNA gene sequencing, library preparation, and phylogenetic tree reconstruction.

16S rRNA gene MiSeq amplicon sequencing was carried out from the 30-to-36-cm-depth interval of triplicate cores. Details on the procedure can be found in Text S1. Qualitative and quantitative information regarding MiSeq sequence reads can be found in Fig. S1 in the supplemental material. Amplification of partial *Geobacter* and *Methanosarcina* 16S rRNA gene sequences was done as described before (76). Cloning employed the TOPO TA cloning kit (Thermo, Fisher Scientific) followed by direct sequencing of PCR products from cloned plasmid DNA (Macrogen). Maximum likelihood phylogenetic trees were constructed using Geneious (77).

### ^13^C labeling experiments.

Cultures were incubated with a 1:9 mix of ^13^CH_3_COOH and unlabeled acetate. Approximatgely 21 cultures with GAC and 16 for the GAC-free cultures were started for the nanoSIMS experiment, because we would sacrificially harvest three at each time point. Headspace gas samples and VFA samples were analyzed as above.

We followed enrichment of ^13^CO_2_ over time by using IR-MS. Briefly, 2.5-ml media samples were retrieved anaerobically for ^13^CO_2_ analyses and immediately stored with 20 µl HgCl_2_-saturated water, without any headspace, and acidified as explained above for DIC analyses in sediment samples; finally, IR-MS analyses were carried out manually against CO_2_ gas standards and bicarbonate standards.

We followed the incorporation of labeled acetate (^13^CH_3_COOH) into a specific phylotype by using CARD-FISH coupled to nanoSIMS, as described below (78).

### CARD-FISH.

To count cells of a specific phylogenetic group and label cells prior to nanoSIMS, we used CARD-FISH as described previously (79) and the following probes: Non338 (80) to check for nonspecific binding, Eub338I-III (81, 82) to target *Eubacteria*, Geo3a-c in equimolar amounts with helpers H-Geo3-3 and H-Geo3-4 to target the *Geobacterales* cluster (83); Arch915 (72) to target *Archaea*, and MS821 (72) to target *Methanosarcina* species. A detailed description of the CARD-FISH protocol can be found in Text S1.

### Quantitative imaging of ^13^C label incorporation via nanoSIMS.

Chemical imaging and quantitative analysis of ^13^C label incorporation was carried out on a NanoSIMS-50L instrument (Cameca, Ametek) operating in negative extraction mode. nanoSIMS analyses were carried out on laser microdissection-selected fields, and the collected data were quantitatively analyzed using the LANS software (84). A detailed description of the protocol used for nanoSIMS analyses and data collection can be found in Text S1.

### Accession number(s).

Sequence files for our partial *Geobacter* and *Methanosarcina* 16S rRNA gene sequences and 16S MiSeq sequence data can be found at NCBI under BioProject ID PRJNA415800.

10.1128/mBio.00226-18.9 TABLE S2  Read count and quality parameters for 16S rRNA gene amplicon sequencing of methanogenic zones (30 to 36 cm) from three Baltic Sea cores at station RA2. *N* (%) indicates the *N*-base percentage in the sequence reads; GC (%) is the GC content of the sequence reads as a percentage; Q20 and Q30 show the percentage bases for which the phred quality score was above 20 or 30, respectively. Download TABLE S2, DOCX file, 0.05 MB. Copyright © 2018 Rotaru et al. 2018 Rotaru et al. This content is distributed under the terms of the Creative Commons Attribution 4.0 International license.
