# Supplementary figures and images for: Conductive Particles Enable Syntrophic Acetate Oxidation between Geobacter and Methanosarcina from Coastal Sediments
Source: mBio. 2018 May 1;9(3):e00226-18. doi: 10.1128/mBio.00226-18 (PMC5930305; doi:10.1128/mBio.00226-18)

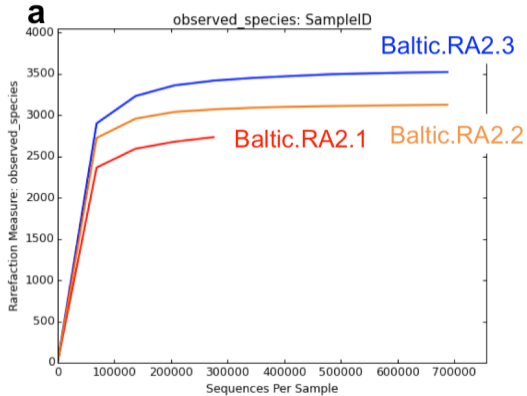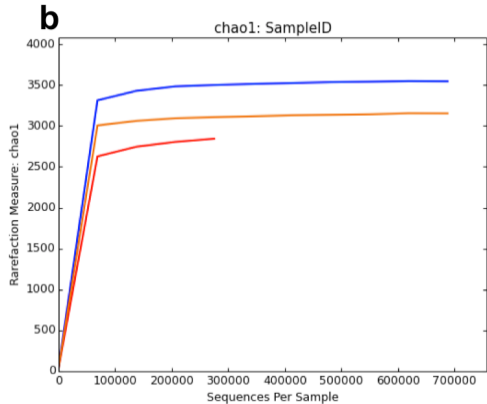

Supplement: FIG S1 [file mbo002183849sf1.pdf]

Deltaproteobacteria

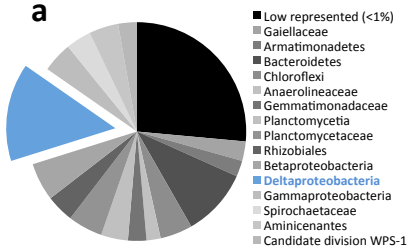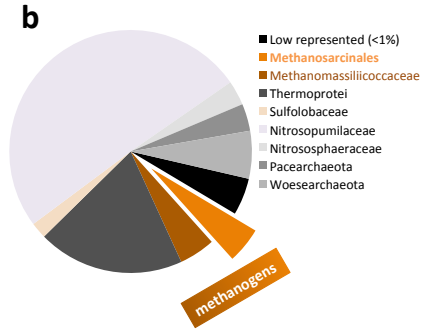

Supplement: FIG S2 [file mbo002183849sf2.pdf]

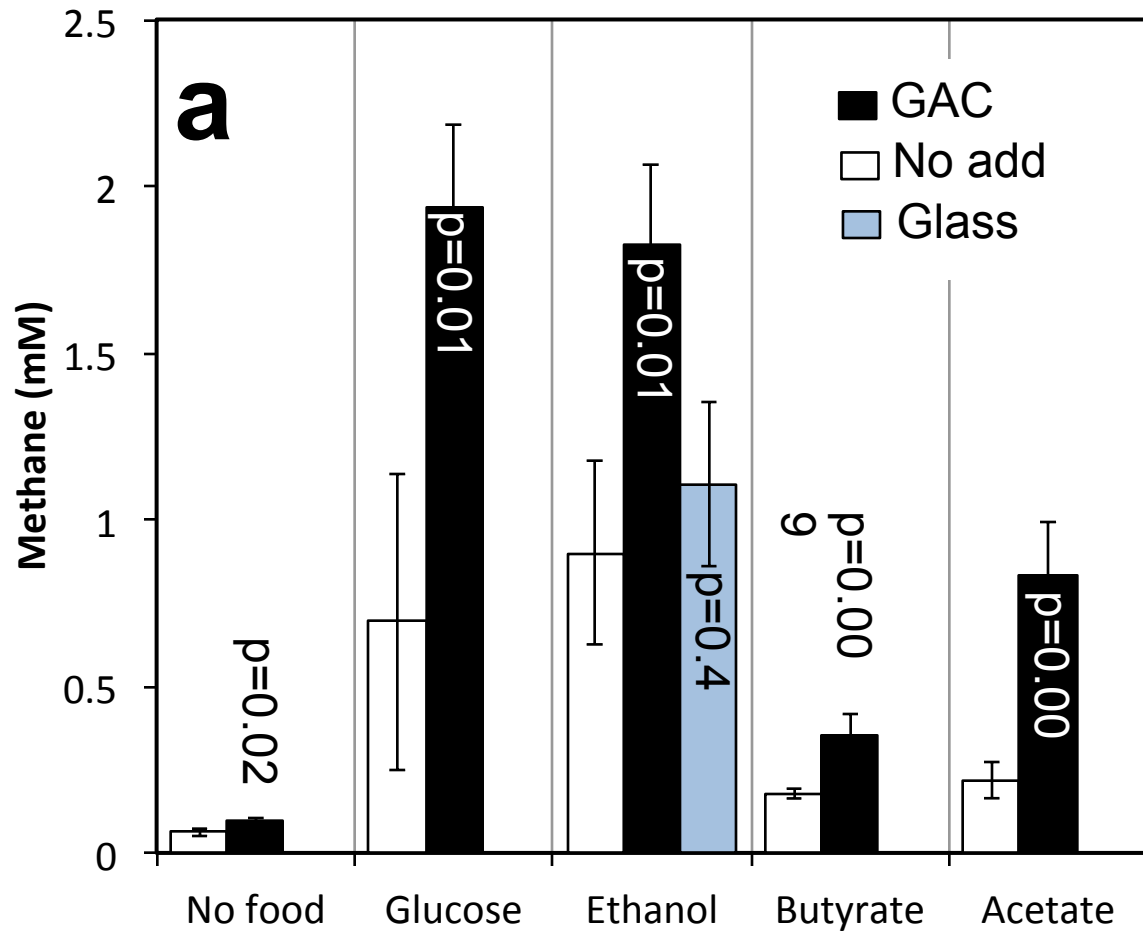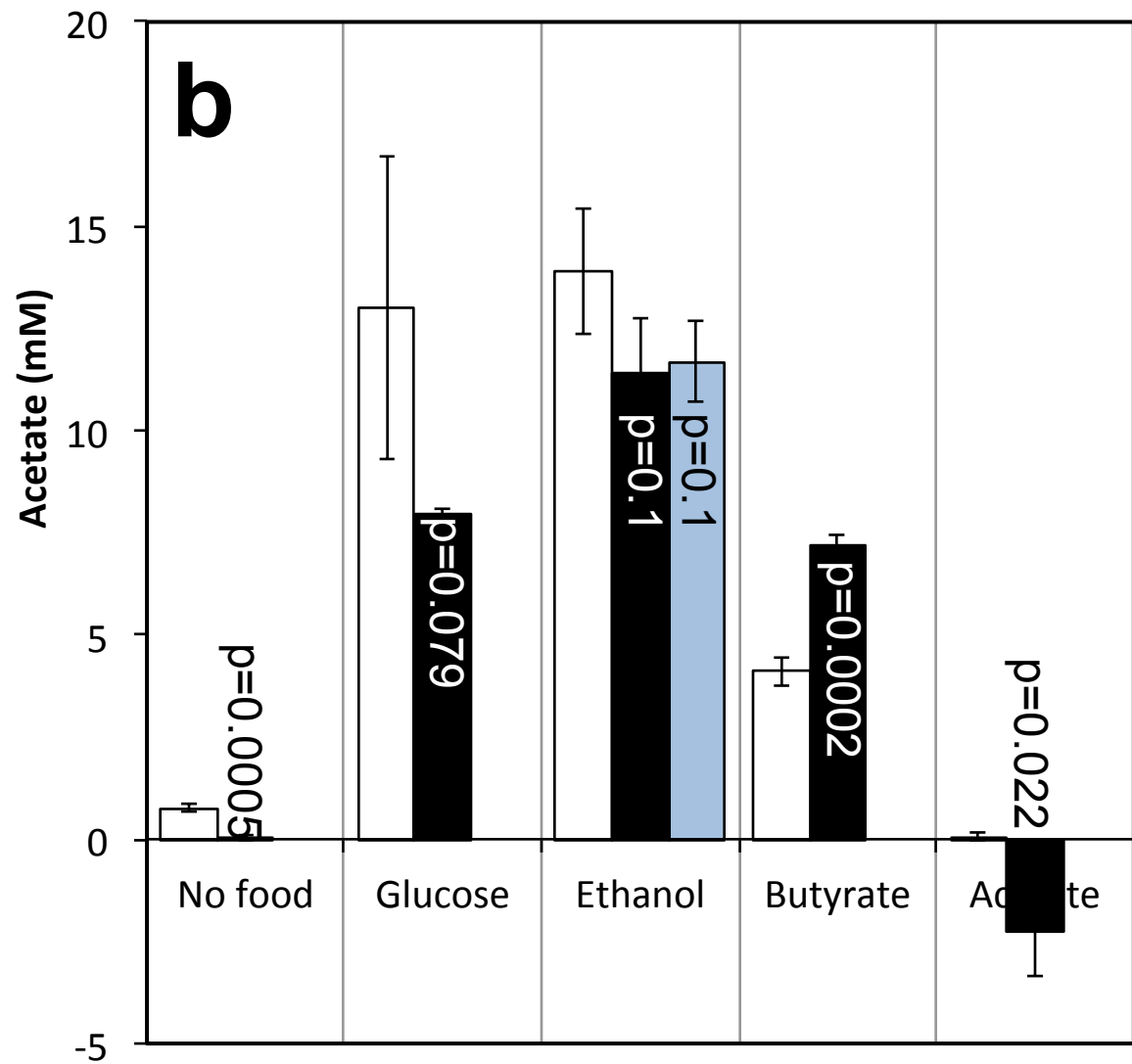

Supplement: FIG S3 [file mbo002183849sf3.pdf]

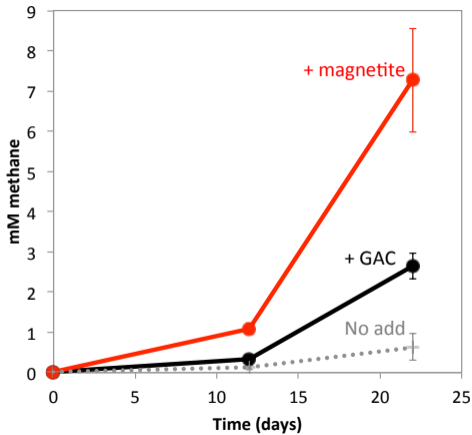

Supplement: FIG S4 [file mbo002183849sf4.pdf]

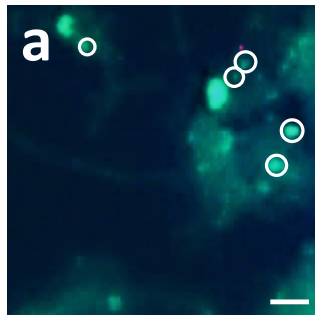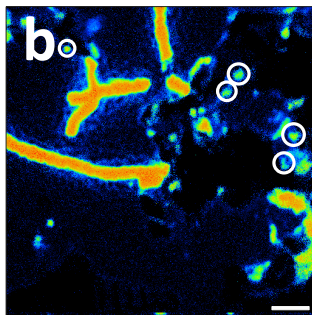

1 2 3 4 5 6  
 $^{13}\text{C}$  fraction[%]

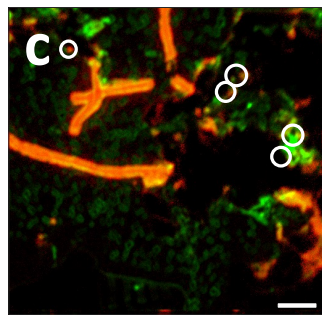

6 Overlay image,  $^{32}\text{S}$  green showing total biomass and  $^{13}\text{C}$  fraction red

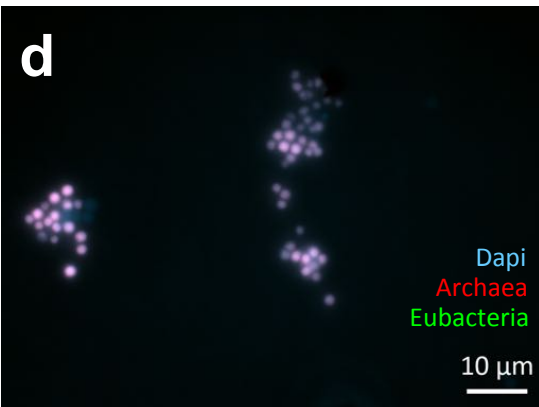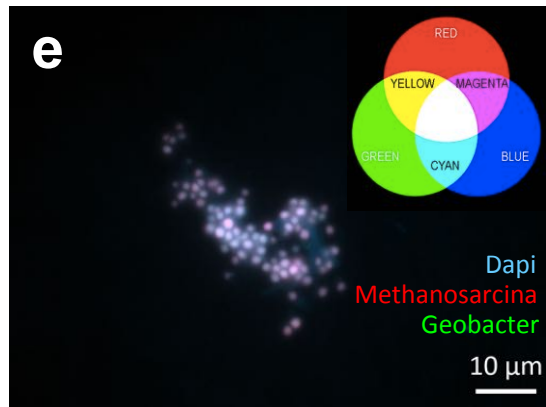

Supplement: FIG S5 [file mbo002183849sf5.pdf]
