# Supplementary material for: Conductive Particles Enable Syntrophic Acetate Oxidation between Geobacter and Methanosarcina from Coastal Sediments
Source: mBio. 2018 May 1;9(3):e00226-18. doi: 10.1128/mBio.00226-18 (PMC5930305; doi:10.1128/mBio.00226-18)
Supplement: FIG S6 [file mbo002183849sf6.pdf]

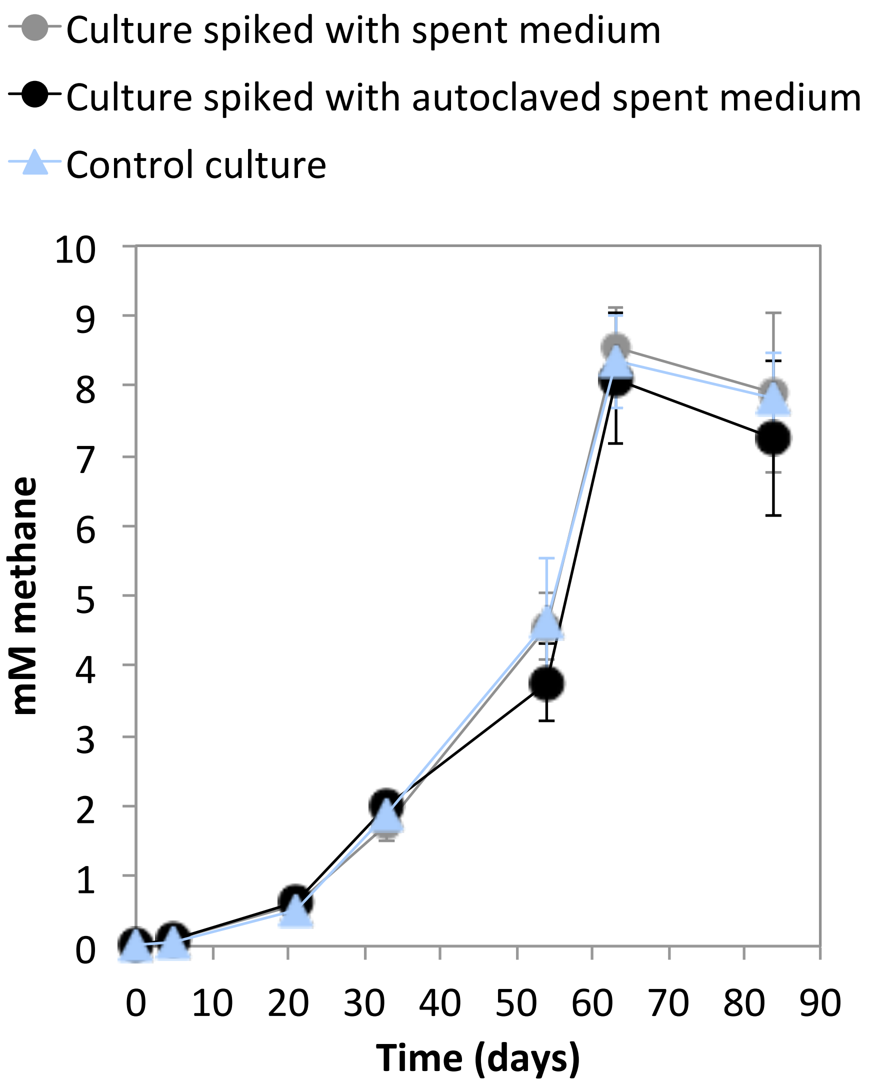

Culture spiked with spent medium
Culture spiked with autoclaved spent medium
Control culture
mM methane
0
1
2
3
4
5
6
7
8
9
10
0
10
20
30
40
50
60
70
80
90
Time (days)
