# Supplementary material for: Conductive Particles Enable Syntrophic Acetate Oxidation between Geobacter and Methanosarcina from Coastal Sediments
Source: mBio. 2018 May 1;9(3):e00226-18. doi: 10.1128/mBio.00226-18 (PMC5930305; doi:10.1128/mBio.00226-18)
Supplement: TEXT S1 [file mbo002183849s1.docx]

**Supplementary methods**

**Conductive particles enable syntrophic acetate oxidation between Geobacter and Methanosarcina from coastal sediments**

**Authors**: Amelia-Elena Rotaru^🖂1^, Hryhoriy Stryhanyuk^2^, Federica Calabrese^2^, Florin Musat^2^, Pravin Malla Shrestha^3^, Hannah Sophia Weber^1^, Oona L.O. Snoeyenbos-West^1^, Per O.J. Hall^4^, Hans H. Richnow^2^, Niculina Musat^🖂2^, Bo Thamdrup^1^

# 16S rRNA gene amplicon sequencing

For amplicon sequencing the following primer pair was used S-D-Arch-0519-a-S-15/ S-D-Bact-0785-b-A-18 which according to Klindworth et al., was best for MiSeq, targeting more than 89% *Bacteria* and more than 88% *Archaea* (1). PCR amplification and indexing (using Nextera XT index kit, Illumina) of the PCR products for all triplicate samples was conducted following the Illumina 16S rRNA gene amplicon sequencing protocol (Illumina, USA). The samples were then pooled in equimolar concentration and were sequenced using ×300 PE MiSeq sequencing at [Macrogen](http://macrogen.com/eng/) (www.macrogen.com), using Illumina’s protocol. The sequences generated (1 to 2 million reads per sample were imported in CD-HIT-OTU to remove noise data and cluster them into OTUs, using a 97% species cutoff (2). For taxonomy

and diversity analysis clean and clustered OTUs were analyzed using QIIMEs bioinformatics pipeline. Alpha rarefraction analyses showed sufficient coverage of the diversity in all three sediment cores, although core 1 had less reads and therefore less OTUs than the other two.

**Quantitative PCR**

**Conditions for PCR:** PCR with specific primers was performed in a final volume of 25µl of which 10µl were 5Prime Hot Master Mix, 0.25µl BSA (stock 10 mg/ml), 1µl or forward and reverse primer (10µM stock each) and 1µl template.

PCR amplification was carried out as follows: 1) hot start at 94°C for 2 minutes, 2) denaturation 1 min at 94°C, 3) 1 min at the annealing temperature appropriate for the primer pair used, as above; and 4) 2 min extension at 72°C. Steps 2 to 4 were repeated 34 times. The amplification was terminated with 10 minutes elongation at 72°C, and stored at 4°C until use. PCR amplicons were checked on a 1.5% agarose gel prepared in TAE buffer.

**Standards for qPCR:** To prepare standards for quantitative PCR we used the primer pairs that produced amplifiable PCR fragments (Table 2_Suppl.File). We purified PCR amplicons in 1.2% agarose prepared in modified TAE buffer. Amplicons were excised under UV light and extracted from agarose with a QIAEX II gel extraction kit following the manufacturer’s protocol. The product is then ligated into pJET1.2/blunt using the protocol provided with the CloneJET PCR cloning kit for sticky end cloning. The ligation mixture was then transformed using the TOPO TA cloning kit for sequencing, following the One Shot Chemical transformation protocol provided by the kit manufacturer. Transformed cells were spread on LB plates with ampicillin (100µg/ml). Colonies were picked and analyzed for insert using the pJET forward and reverse primers according to the CloneJET protocol. Successful colonies were reamplified, gel purified on 1.2% agarose gel in modified TAE, extracted from gel by QIAEXII, quantified by NanoDrop and diluted from 10^9^ copies to 10^0^ copies. These were used as standards for qPCR.

qPCR of environmental samples was always run alongside standards prepared as explained above. We noticed that a ten-fold dilution of the DNA extracted from sediment and enrichments was required for best qPCR results. Dilution of the template worked successfully in minimizing inhibitory effects of sample matrix constituents, as observed by Lloyd et al. when doing qPCR on DNA extracted from various sediment samples (3)

**CARD-FISH procedure**

Samples were fixed for 2 hours at room temperature with 2% formaldehyde solution, and filtered onto 0.2 µm pore size polycarbonate filters (GTTP, 25 mm; Millipore). Filters were dipped in agarose 0.1% (w/w), and permeabilized in lysozyme 10 mg/ml (Sigma-Aldrich, St Louis, MO, USA), at 37ºC for 1 hour, followed by achromopeptidase treatment (60 U mLl-1, 0.01 M NaCl, 0.01 M Tris-HCl, pH 7.6, Sigma-Aldrich, St Louis, MO, USA) for 30 min at 37ºC, for *Eubacteria*, and with SDS 0.5%, 10 min at room temperature, followed by proteinase K (15 µg/ml) treatment for 5 min. at room temperature for *Archaea* and *Methanosarcina*.

Hybridization was carried out at 46ºC for 3 h using the Horseradish peroxidase (HRP) labeled probes (50 ng µL^-1^).

The Horseradish peroxidase (HRP) labeled probes (50 ng µLl-1) (http://www.biomers.de) were diluted in 900 µL of hybridization buffer with the corresponding formamide (FA) concentration for each probe: 35% FA for Arch915, Eub338I-III and Non338, 30% for Geo3a-c and 40% for MS821 probes. Fluorescently-labelled tyramide Alexa Fluor® 488 (1 mg mLl-1, 46ºC, 20 min, ThermoFisher, Germany) was used for the amplification step. The hybridized cells were further stained with 4´,6-diamidino-2-phenylindole (DAPI) at 1 µg mLl-1 and were quantified using an Epifluorescence microscope Axio Imager.Z2 from Carl Zeiss (Zeiss, Germany).

Counts of hybridized bacteria (minimum of 1000 DAPI-stained cells were showed as means calculated from 10 randomly chosen microscopic fields as percentage of total DAPI stained cells. Whenever CARD-FISH was performed prior to NanoSIMS, we used gold-palladium sputtered polycarbonate filters (type GTTP; pore size, 0.22 µm; diameter, 25 mm; Millipore, Eschborn, Germany) as conductive support for the cells. A laser dissection microscope (LMD) in PALM IV CombiSystem based on Axio Observer Microscope from Carl Zeiss (Zeiss, Germany) was used to select and mark regions of interest containing the target hybridized cells for the following NanoSIMS analysis.

**NanoSIMS procedure**

During chemical imaging and quantitative analysis of ^13^C label incorporation by NanoSIMS-50L, a 2 pA DC beam of 16 keV Cs^+^ ions was focused in a 70 nm spot at the sample surface analyzing 30x30 µm² areas in 512x512 px raster with 2 msec dwell time per pixel. Before the analysis, the sample surface of 100x100 µm² area was treated with 12 nA of low-energy (50 eV) Cs^+^ beam for 10 minutes. The low-energy deposition of caesium has been performed with the purpose to equilibrate the working function for negative secondary ions and to make the outermost layer of the sample available for the analysis avoiding its sputtering during high-energy implantation with 16 keV Cs^+^ beam. The secondary ion species were analyzed for their mass and charge ratio (m/z) using the seven available detectors as follows: ^12^C^-^ (detector-1), ^13^C^-^ (detector-2), ^16^O^-^ (detector-3), ^12^C^14^N^-^ (detector-4), ^13^C^14^N^-^ (detector-5), ^31^P^-^ (detector-6), ^32^S^-^ (detector-7). The mass resolving power (MRP=M/dM) was checked to be between 7000 and 12000 with the exit slit width of 40 µm, 20 µm wide entrance slit, 200 µm aperture slit and with the energy slit cutting about 30% of secondary ions in high-energy tail of their energy distribution. The microbial cells have been shown to be sputtered completely after 55 scans in average upon the used preimplantation and analysis conditions. Therefore the data planes acquired with scans 3 to 50 were accumulated and considered for the analysis employing LANS software (4) allowing for lateral drift correction and quantitative analysis of isotope ratio (^13^C^14^N/^12^C^14^N and ^13^C/^12^C). Quantitative analysis of ^13^C incorporation was based on ^13^C^14^N/^12^C^14^N ratio to avoid the reduction of calculated ^13^C fraction due to the ^12^C originating from cell surroundings and embedding agarose as it was revealed for ^13^C/^12^C ratio.

**Carbon density measurements by Elemental Analyzer Isotope Ratio** **Mass Spectrometery (EA-IRMS) for assimilation estimates using NanoSIMS**

Carbon density per cell was measured for two representative strains: *Methanosarcina honorebensis (DSMZ no or ATTC)* and *Geobacter metallireducens* GS15. *M. horonobensis* was preferred because it does not aggregate so we could determine C-density per cell.

Cultures were grown in under strictly anaerobic conditions as described before (5).

In order to determine the carbon density per cell we withdrew 2 different volumes from each culture. The carbon measurement was done with an Elemental Analyzer (EA) isotope ratio mass spectrometer (IRMS). Different volumes of 4 ml and 5 ml, respectively of each culture corresponding to the two strains s were filtered onto pre-combusted (450^o^C) 0.45 μm pore size GF/F filters (Whatman™, GE Healthcare) using vacuum filtration manifold (Millipore® model 1225, Sigma Aldrich) and washed three times with 5 ml of ddH_2_O. Filters were first air dried at Room Temperature (RT) inside a biosafety cabinet and then exposed to hydrochloric acid vapor (HCl 20%) overnight inside a desiccator. Before EA analysis, we excised 5 mm diameter (Ø) filter pieces (two pieces per each GF/F filter) using a sterilized hollow punch. Filter pieces were packed in 3.5 x 5 mm diameter Zinc cups (Hekatech) and loaded on the auto sampler carousel of the EA-IRMS. As laboratory standard we used the following quantities of sucrose: 0.038 mg, 0.050 mg, 0.080 mg, 0.124 mg, 0.150 mg, 0.164 mg, 0.186 mg, 0.230 mg.

Parallel filters for cell counting were prepared from each culture as follows: a volume of 500 μl was mixed with 500 μl of 2% Paraformaldehyde (PFA) in Phosphate Buffer Saline (PBS 1X). 10 μl of this mixture was filtered onto 0.2 µm pore size polycarbonate filters (GTTP, 25 mm; Millipore, Eschborn, Germany using a steel syringe filter holder (Sartorius, 16214) and nitrocellulose filters 0.45 μm pore size as support filters. For Methanosarcina culture, the suspension was mild sonicated (MS73, Sonupuls UW70, Bandelin, Berlin, Germany) in order to disperse the cells better for the filtration step (3 cycles of , 2´´ pulses, 2´´ pause at 50% power).

After filtration, the cells were washed with PBS 1X and dehydrated with 50%, 70% and 80% ethanol (in ddH2O), respectively,1 min each step. Filters were air dried and stained with 4’6’-diamidino-2-phenylindole (DAPI) (1 µg mL^-1^) (20 minutes in the dark at RT). Filters were mounted using Citifluor:Vectashield (80:20) mix and stored at -20°C until fluorescence microscopy observation. Further the counting was done using optical fluorescence microscope (Axio Imager.Z2) and Zen software (Zeiss) for evaluation of the images. ). 10 to 15 randomly chosen microscopic fields along the filter length were counted for each filter containing 186 to 548 and 145 to 225 DAPI stained cells of *Geobacter metallireducens* and of *Methanosarcina horonobensis*, respectively. The number of cells/ml was calculated taking into account the average value of the DAPI counts for each strain and the dilutions used from the original sample: 0.5 dilution with 2% PFA, and 0.01 for the volume (μl) filtered.

Using the number of cells ml^-1^ we calculated the carbon density cell^-1^. This number was calculated taking into account the initial filtered volume (4 mL or 5mL) and the diameter of the analyzed area (5mm Ø). We further calculated the carbon density μm^-3^ (fg C μm^-3^) by dividing the C density cell^-1^ to the biovolume.

**The biovolume** corresponding to each culture was calculated from scanning electron (SEM) micrographs. 18 cells of each strain were measured until the moving average stabilized (data not shown). The moving average of biovolumes for *Geobacter metallireducens* and *Methanosarcina barkeri* cells from SEM images of the two organisms grown together in coculture for a previous study (48). Fanghua Liu took SEM images of the cells in 2013. We calculated that *Geobacter metallireducens* has a biovolume of 0.385±0.133 µm^3^ while *Methanosarcina* biovolume ranges about 1.809±0.719 µm^3^. We used the following formula (V_cilinder_ + V_hemisphere1_+ V_hemisphere2_) to calculate the biovolume of Geobacter cells, where V_cylinder_ is π r^2^ h, and the V_hemisphere_ is 2/3 π r^3^, and just the V_sphere_ 4/3 π r^3^ to calculate the biovolume of *Methanosarcina*.

**NanoSIMS calculations**: Calculation was based on single-cell assimilation rates from NanoSIMS, as average, the total cell number in bottles (considered for 10 ml culture), carbon density per cell and call type and their specific biovolume.

A simplified formula for assimilation in each cell type is:

$$\frac{Assimilation per cell \times C-density of a cell type}{Biovolume of a cell type}\times cell numbers$$

Note that we cannot calculate as percentage of total assimilated acetate because we can’t determine accurately assimilation in ‘unidentified cells’ since we don’t know the exact abundance of each type of ‘unidentified cells’ in the total population.

1. Klindworth A, et al. (2013) Evaluation of general 16S ribosomal RNA gene PCR primers for classical and next-generation sequencing-based diversity studies. *Nucleic Acids Res* 41(1):1–11.

2. Li W, Fu L, Niu B, Wu S, Wooley J (2012) Ultrafast clustering algorithms for metagenomic sequence analysis. *Brief Bioinform* 13(6):656–668.

3. Lloyd KG, MacGregor BJ, Teske A (2010) Quantitative PCR methods for RNA and DNA in marine sediments: Maximizing yield while overcoming inhibition. *FEMS Microbiol Ecol* 72(1):143–151.

4. Polerecky L, et al. (2012) Look@NanoSIMS - a tool for the analysis of nanoSIMS data in environmental microbiology. *Environ Microbiol* 14(4):1009–1023.

5. Rotaru A-E, et al. (2014) Direct interspecies electron transfer between Geobacter metallireducens and Methanosarcina barkeri. *Appl Environ Microbiol* 80(15):4599–605.

**References**

1. Klindworth A, et al. (2013) Evaluation of general 16S ribosomal RNA gene PCR primers for classical and next-generation sequencing-based diversity studies. *Nucleic Acids Res* 41(1):1–11.

2. Li W, Fu L, Niu B, Wu S, Wooley J (2012) Ultrafast clustering algorithms for metagenomic sequence analysis. *Brief Bioinform* 13(6):656–668.

3. Lloyd KG, MacGregor BJ, Teske A (2010) Quantitative PCR methods for RNA and DNA in marine sediments: Maximizing yield while overcoming inhibition. *FEMS Microbiol Ecol* 72(1):143–151.

4. Polerecky L, et al. (2012) Look@NanoSIMS - a tool for the analysis of nanoSIMS data in environmental microbiology. *Environ Microbiol* 14(4):1009–1023.

5. Rotaru A-E, et al. (2014) Direct interspecies electron transfer between Geobacter metallireducens and Methanosarcina barkeri. *Appl Environ Microbiol* 80(15):4599–605.
