# Supplementary material for: Conductive Particles Enable Syntrophic Acetate Oxidation between Geobacter and Methanosarcina from Coastal Sediments
Source: mBio. 2018 May 1;9(3):e00226-18. doi: 10.1128/mBio.00226-18 (PMC5930305; doi:10.1128/mBio.00226-18)
Supplement: TABLE S1 [file mbo002183849st1.docx]

**Table 1SF**

List of primers used in this study.

| Primer pair | Sequence (5’-3’) | Target 16S (specificity %)^1^ | Ref |
| --- | --- | --- | --- |
| S-D-Arch-0519-a-S-15  S-D-Bact-0785-b-A-18 | CAGCMGCCGCGGTAA  TACNVGGGTATCTAATCC | *Bacteria* (89%) +  *Archaea* (88%) | (1) |
| Geo494F  Geo825R | AGGAAGCACCGGCTAACTCC  TACCCGCRACACCTAGT | *Geobacteraceae* (96%) +  *Desulfuromonadaceae* (100%) | (2) |
| Rho471F  Rho830R | GGGCTAATGACGGTACCGTA  CCAGTTGACATCGTTTAGGG | *Rhodoferax ferrireducens* (100%)  *Rhodoferax* (67%) | (3) |
| SW 783 F  SW 1245 R | AAAGACTGACGCTCAKGCA  TTYGCAACCCTCTGTACT | *Shewanella* (28%)  *Shewanella baltica* (100%) | (4) |
| GX 182 F  GX 472 R | AGACCTTCGGCTGGGATGCT  AGGTACCGTCAAGTAACASS | *Geothrix* (100%) | (4) |
| MSC 380 F  MSC 828 R | GAAACCGYGATAAGGGGA  TAGCGARCATCGTTTACG | *Methanosarcinaceae* (72%) | (5) |
| MCC 495 F  MCC 832 R | TAAGGGCTGGGCAAGT  CACCTAGTYCGCARAGTTTA | *Methanococcaceae* (100%)  *Methanocaldococcus* (71%) | (5) |
| MBT 857 F  MBT 1196 R | CGWAGGGAAGCTGTTAAGT  TACCGTCGTCCACTCCTT | *Methanobacteriaceae* (93%) | (5) |
| MMB 282 F  MMB 832 R | ATCGRTACGGGTTGTGGG  CACCTAACGCRCATHGTTTAC | *Methanomicrobiales* (86%) | (5) |
| 8F  Geo825R*a* | AGAGTTTGATYMTGGCTCAG  TACCCGCRACACCTAGTTCT | *Geobacter psychrophilus* (100%) | (4) |
| Arch344F  Arch915R | ACGGGGYGCAGCAGGCGCGA  GTGCTCCCCCGCCAATTCCT | *Euryarachaeota* (90%) | (6) |
| ^1^Specificity for the genus/species-specific primers was tested against the Silva LTP (living tree taxonomy) database of 16S from cultured microorganisms. | | | |

**References**

1. Klindworth A, et al. (2013) Evaluation of general 16S ribosomal RNA gene PCR primers for classical and next-generation sequencing-based diversity studies. *Nucleic Acids Res* 41(1):1–11.

2. Holmes DE, Finneran KT, O’Neil RA, Lovley DR (2002) Enrichment of members of the family Geobacteraceae associated with stimulation of dissimilatory metal reduction in uranium-contaminated aquifer sediments. *Appl Environ Microbiol* 68(5):2300–2306.

3. Kim S-J, et al. (2012) Molecular analysis of spatial variation of iron-reducing bacteria in riverine alluvial aquifers of the Mankyeong River. *J Microbiol* 50(2):207–217.

4. Snoeyenbos-West OL, Nevin KP, Anderson RT, Lovley DR (2000) Enrichment of Geobacter species in response to stimulation of Fe(III) reduction in sandy aquifer sediments. *Microb Ecol* 39(2):153–167.

5. Yu Y, Lee C, Kim J, Hwang S (2005) Group-specific primer and probe sets to detect methanogenic communities using quantitative real-time polymerase chain reaction. *Biotechnol Bioeng* 89(6):670–679.

6. Raskin L, Stromley JM, Rittmann BE, Stahl DA (1994) Group-specific 16S rRNA hybridization probes to describe natural communities of methanogens. *Appl Environ Microbiol* 60(4):1232–40.
