# Supplementary material for: Conductive Particles Enable Syntrophic Acetate Oxidation between Geobacter and Methanosarcina from Coastal Sediments
Source: mBio. 2018 May 1;9(3):e00226-18. doi: 10.1128/mBio.00226-18 (PMC5930305; doi:10.1128/mBio.00226-18)
Supplement: TABLE S2 [file mbo002183849st2.docx]

**Table 2SF**

Read count and quality parameters for 16S rRNA gene amplicon sequencing of methanogenic zone (30-36cm) from three Baltic Sea cores at station RA2. N(%) indicates the N-base percentage in the sequence reads. GC(%) is the GC content of the sequence reads in percentage. Q20 and Q30 show the percentage bases for which the phred quality score is above 20 or 30 respectively.

| Result of Merge (by FLASH) | | | | | | |
| --- | --- | --- | --- | --- | --- | --- |
| Sample Name | Total Bases | Read Count | N (%) | GC (%) | Q20 (%) | Q30 (%) |
| Core RA2.1 | 311,020,958 | 1,098,752 | 0 | 54.67 | 94.94 | 89.57 |
| Core RA2.2 | 588,089,281 | 2,078,323 | 0 | 54.72 | 96.78 | 93.19 |
| Core RA2.3 | 513,699,067 | 1,816,422 | 0 | 54.97 | 97.14 | 93.98 |
